# The effect of maternal and fetal weight on the risk of emergency cesarean section in nulliparous women

**DOI:** 10.1097/MD.0000000000041095

**Published:** 2025-01-17

**Authors:** Jing Bao, Ping Guan

**Affiliations:** a Department of Obstetrics, Maternal and Child Health Hospital of Hubei Province, Wuhan, China.

**Keywords:** emergency cesarean section, gestational weight gain, maternal weight, prepregnancy body mass index

## Abstract

Although many studies based on different ethnic groups have analyzed the impact of maternal and infant weight on overall cesarean section rates in recent years, research on the impact of maternal and infant weight on emergency cesarean section (EmCS) rates is lacking, especially in the Chinese population. This study aimed to analyze whether maternal and fetal weight could influence the risk of EmCS. A total of 8427 nulliparous women who delivered vaginally (full-term, singleton, and cephalic presentation) were included in this study and divided into the normal vaginal delivery (VD) and EmCS groups. Of 8427 cases, 909 (10.8%) were delivered by EmCS because of failed VD. Compared with pregnant women with a normal body mass index, the risk of EmCS in overweight women increased significantly (*P* < .001). Birth weight > 3550 g was associated with an increased risk of EmCS. Subgroup analyses showed that among women with underweight and normal weight, old age, inadequate gestational weight gain, and large for gestational age were independent high-risk factors for EmCS (*P* < .05), whereas small for gestational age was the low-risk factor. Compared with the fetal distress group, the weight of newborns in the nonfetal distress group was significantly higher (*P* < .001), and the main cause of EmCS in women with macrosomia, large for gestational age, or birth weight ≥ 3550 g was fetal distress (*P* < .05). The prepregnancy maternal and fetal weights can affect the risk of EmCS. Weight management should be enhanced to control gestational weight gain according to the prepregnancy body mass index to reduce the risk of EmCS due to failed VD.

## 1. Introduction

In recent years, the incidence of overweight/obesity has been increasing in both developed and developing countries and is becoming a worldwide health problem.^[1,2]^ Currently, the global epidemic of overweight/obesity has led to maternal hyperglycemia, affecting 1 in every 6 pregnancies worldwide, prompting intense research interest in maternal weight management.^[3]^ Obesity and excessive gestational weight gain (GWG) increase the soft tissues of mothers and the rate of macrosomia, resulting in serious situations such as shoulder dystocia, in which mothers and babies are at risk.^[4,5]^ Neonatal birth weight is also on the rise, and the average birth weight of full-term newborns in China over the past 30 years has increased by 159 g.^[6]^ Thus, abnormal weight in pregnant women and infants is associated with perinatal complications such as hypertensive disorders of pregnancy (HDP) and abnormal liver function, which leads to a series of adverse maternal and infant outcomes, including an increase in the rate of cesarean section (CS).^[7,8]^ Global rates of CS have increased to some extent.^[9,10]^ Compared with normal vaginal delivery (NVD), excessive usage of CS may be harmful to both mothers and children.^[11,12]^ Some studies suggest that controlling prepregnancy maternal and infant weights may control the occurrence of definite CS indications, such as preeclampsia and eclampsia, which can effectively reduce the CS rate and perinatal complications.^[13]^ However, most previous studies did not estimate the performance of the 2 CS procedures separately: emergency CS (EmCS) and elective CS (ElCS).^[14,15]^ In fact, it should be noted that there are some special populations without clear indications for ElCS before delivery, but EmCS is needed due to the failure of vaginal delivery (VD). EmCS is a special kind of CS that is conducted in emergence, with indications including, but not limited to, fetal distress (FD), relative head-pelvic imbalance, and failed induction of labor.^[16]^ Women with EmCS have been found to have higher maternal and infant complications than those who undergo ElCS.^[17]^ If EmCS is not performed in time, delivery cannot be terminated within a limited time and may result in maternal and infant death.^[18]^ Therefore, paying attention to EmCS is more conducive to control adverse pregnancy outcomes. Although many studies based on different ethnic groups have analyzed the impact of maternal and infant weight on overall CS in recent years, there is a lack of research on the impact of maternal and infant weight on the EmCS rate, especially in the Chinese population.

This study was designed to include nulliparous women with a singleton cephalic presentation within 37 to 41 + 6 weeks of gestation with an uncomplicated pregnancy who attempted VD as the study population and to analyze the influencing factors of EmCS, especially the effect of maternal and fetal weight on EmCS rate.

## 2. Materials and methods

### 2.1. Study population

**Figure 1.**
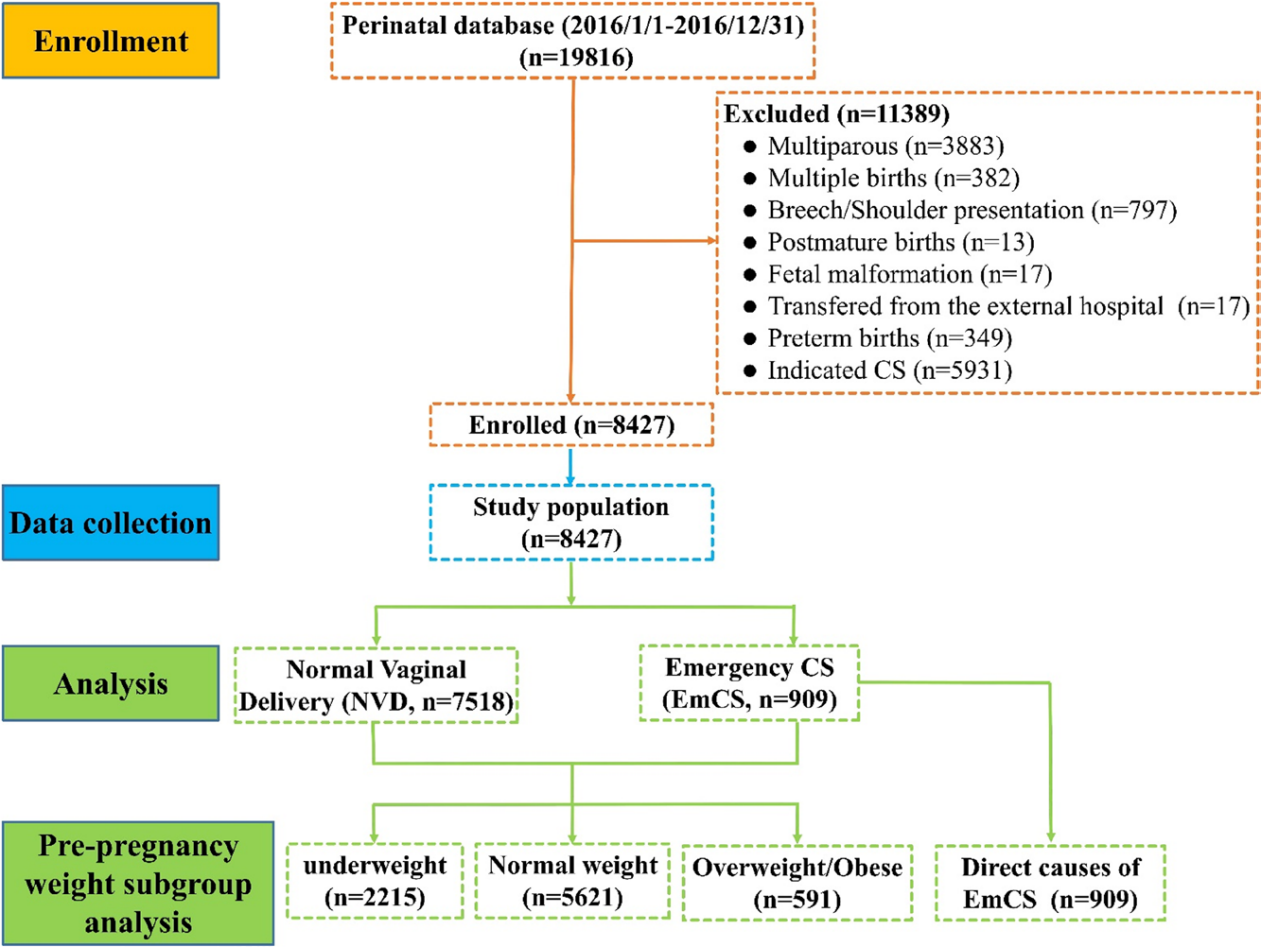
The flowchart of this study. CS = cesarean section, EmCS = emergency cesarean section, NVD = normal vaginal delivery.

In this study, we retrospectively analyzed the information of pregnant women who delivered at Hubei Maternal and Child Health Hospital of Hubei Province between January 1, 2016, and December 31, 2016. Continuous data collection was not performed during the study period. Inclusion criteria include the following: all pregnant women were delivered in the hospital from January 1, 2016, to December 31, 2016; the vaginal examination was performed before VD, and there were no abnormalities in pelvic measurements, complications endangering the lives of mothers and fetuses; and there was no FD and no obvious head-pelvic disproportion before the trial of VD. There were 19,816 cases that met the inclusion criteria. The exclusion criteria are given as follows: multiparous, multiple births, breech or shoulder presentation, CS after full-term pregnancy owing to severe complications of fetal malformation, postterm birth, patients who failed VD in the external hospital were transferred, direct ElCS with certain indications, and preterm birth. According to VD outcomes, all eligible women were classified into 2 groups: the NVD group and the EmCS group. Finally, 8427 nulliparous women with singleton cephalic presentation at 37 to 41^+6^ weeks of gestation with an uncomplicated pregnancy were enrolled in this study, including 7518 in the NVD group and 909 in the EmCS group (Fig. 1). EmCS was defined as cases in which the VD process was discontinued due to certain indications, such as the occurrence of FD, abnormal fetal position, uterine rupture, and presentation of the umbilical cord.

The study protocol was approved by the Medical Ethics Committee of the Maternal and Child Health Hospital of the Hubei Province. The methods were performed in accordance with relevant guidelines and regulations. Because it was a retrospective study, we only extracted clinically relevant information, which did not affect the treatment and health of patients, and the patients’ privacy was protected. Therefore, written informed consent was not applicable for this study. The ethics committee approved the waiver of consent.

### 2.2. Outcome measures

All women included in this study were categorized by the recently developed Chinese body mass index (BMI) standard as follows: underweight (BMI < 18.5 kg/m^2^), normal weight (18.5 kg/m^2^ ≤ BMI < 24.0 kg/m^2^), overweight (24 kg/m^2^ ≤ BMI < 28.0 kg/m^2^), and obese (BMI ≥ 28.0 kg/m^2^). Because there were only 10 obese women, we integrated overweight and obesity into the same group (BMI ≥ 24.0 kg/m^2^). Maternal GWG was recorded as weight gain from prepregnancy to the time immediately before delivery. Three categories, inadequate, adequate, and excessive, were defined according to the updated Institute of Medicine guidelines.^[19]^ Detailed information can be obtained from our previous study.^[20]^

The main end point of this study was the effect of maternal and fetal weight on the EmCS rate, followed by the direct cause of VD failure resulting from abnormal maternal and fetal weights. The variables for synergistic analysis in this study included maternal age, gestational age, maternal height, weight before pregnancy, BMI before pregnancy, weight at delivery, BMI at delivery, GWG, birth weight, small for gestational age (SGA), large for gestational age (LGA), fetal sex, abnormal amniotic fluid characteristics and quantity, umbilical cord status, HDP and induction of labor. Amniotic fluid characteristics are a convenient method to reflect meconium-stained amniotic fluid. In China, meconium-stained amniotic fluid is classified into 3 types: type I, the amniotic fluid appears light green or yellow; type II, the amniotic fluid is green or yellow, turbid, and contains particulate matter; and type III, brownish-yellow, viscous like pea soup. In addition, brown amniotic fluid is considered a marker of intra-amniotic bleeding (bloody).^[21]^ Abnormal umbilical cord included cord entanglement and torsion of the umbilical cord. An abnormal quantity of amniotic fluid includes hypamnion and hydramnion. To cover more cases, we analyzed the impact of fetal weight on EmCS using a 75-percentile to divide women into 2 groups. SGA and LGA were defined as newborns whose birth weights were <10th and >90th percentiles for gestational age, respectively. SGA and LGA were determined by Chinese criteria.^[22]^

### 2.3. Statistical analysis

Quantitative variables were expressed as mean ± SD. Qualitative variables were expressed as absolute and relative frequencies. The *t* test, the χ^2^ test, and the logistic regression analysis were performed using SPSS software, version 20.0, to explore the relationship between the EmCS rate and perinatal factors. Multiple logistic regression analysis was performed in a stepwise manner to identify the factors independently associated with the risk of EmCS. Statistical significance was set at *P* < .05.

## 3. Results

### 3.1. Baseline characteristics of the study population

Of the 8427 nulliparous singleton pregnancies, 7518 (89.2%) were successfully managed with VD and 909 (10.8%) were delivered by EmCS. The demographic and baseline characteristics of each group are summarized in Table 1. The average age of the pregnant women in the EmCS group was significantly higher than that in the NVD group (*P* < .001). Maternal body weight before pregnancy, BMI before pregnancy, maternal weight at delivery, and BMI at delivery were significantly higher in the EmCS group than in the NVD group (*P* value was <.001, .001, <.001, and <.001, respectively). The proportion of underweight women in the EmCS group (22.7%) was significantly lower than that in the NVD group (26.7%; *P* = .001). There were 1254 (14.9%), 2781 (33.0%), and 4392 (52.1%) women with insufficient GWG, adequate GWG, and excessive GWG, respectively. The proportion of women with excessive GWG in the EmCS group (59.8%) was significantly higher than that in the NVD group (51.2%); the difference was statistically significant (*P* < .001).

**Table 1:** Baseline characteristics of the NVD and EmCS groups.

Variables	Study population (n = 8427; %)	Groups		
		NVD (n = 7518; 89.2%)	EmCS (n = 909; 10.8%)	*P* value
Age, yr	27.51 ± 3.03	27.42 ± 3.02	28.30 ± 2.88	<.001
<25	1155 (13.7)	1084 (14.4)	71 (7.8)	<.001
25–29	5379 (63.8)	4820 (64.1)	559 (61.5)	
30–35	1754 (20.8)	1504 (20.0)	250 (27.5)	
≥35	139 (1.6)	110 (1.5)	29 (3.2)	
Height, cm	161.65 ± 0.04	161.64 ± 0.04	161.74 ± 0.04	.508
Gestational age, wk	39.64 ± 1.00	39.65 ± 0.99	39.58 ± 1.05	.057
Weight before pregnancy, kg	51.28 ± 5.29	51.08 ± 5.15	52.88 ± 6.11	<.001
BMI before pregnancy, kg/m^2^	19.64 ± 2.88	19.56 ± 1.98	20.25 ± 2.52	.001
Underweight (<18.5)	2215 (26.3)	2009 (26.7)	206 (22.7)	
Normal weight (18.5–23.9)	5621 (66.7)	5014 (66.7)	607 (66.8)	
Overweight/obese (≥24.0)	591 (7.0)	495 (6.6)	96 (10.6)	
Weight at delivery, kg	69.62 ± 8.05	68.37 + 7.9	70.65 + 8.73	<.001
BMI at delivery, kg/m^2^	26.26 ± 2.88	26.16 ± 2.7	27.04 ± 3.4	<.001
GWG, kg	17.34 ± 5.61	17.29 ± 5.58	17.76 ± 5.81	.016
Inadequate	1254 (14.9)	1098 (14.6)	156 (17.2)	<.001
Adequate	2781 (33.0)	2572 (34.2)	209 (23.0)	
Excessive	4392 (52.1)	3848 (51.2)	544 (59.8)	
Birth weight, g	3315.33 ± 366.92	3295.61 ± 359.36	3478.41 ± 387.81	<.001
<2500	49 (0.6)	42 (0.6)	7 (0.8%)	<.001
2500–4000	8026 (95.2)	7218 (96.0)	808 (88.9)	
≥4000	352 (4.2)	258 (3.4)	94 (10.3)	
75-percentile of birth weight, g				
<3550	6145 (72.9)	5644 (75.1)	501 (55.1)	<.001
≥3550	2282 (27.1)	1874 (24.9)	408 (44.9)	
Fetal growth				
SGA	1439 (17.1)	1359 (18.1)	80 (8.8)	<.001
AGA	6045 (71.7)	5427 (72.2)	618 (68)	
LGA	943 (11.2)	732 (9.7)	211 (23.2)	
Fetal gender				
Male	4324 (51.3)	3812 (50.7)	512 (56.3)	.001
Female	4103 (48.7)	3706 (49.3)	397 (43.7)	
Characters of amniotic fluid				
Clear	6020 (71.4)	5582 (74.2)	438 (48.2)	<.001
Abnormal	2407 (28.6)	1936 (25.8)	471 (51.8)	
Umbilical cord				
Normal	5256 (62.4)	4774 (63.5)	482 (53.0)	<.001
Abnormal	3171 (37.6)	2744 (36.5)	427 (47.0)	
HDP				
No	8063 (95.7)	7216 (96.0)	847 (93.2)	<.001
Yes	364 (4.3)	302 (4.0)	62 (6.8)	
Abnormal quantity of amniotic fluid				
No	8339 (99.0)	7472 (99.4)	867 (95.4)	<.001
Yes	88 (1.0)	46 (0.6)	42 (4.6)	
Induction of labor				
No	7289 (86.5)	6710 (89.3)	579 (63.7)	<.001
Yes	1138 (13.5)	808 (10.7)	330 (36.3)	

AGA = appropriate for gestational age, BMI = body mass index, EmCS = emergency cesarean section, GWG = gestational weight gain, HDP = hypertensive disorders of pregnancy, LGA = large for gestational age, NVD = normal vaginal delivery, SGA = small for gestational age.

The mean birth weight of the EmCS group was significantly higher than that of the NVD group (*P* < .001). The 75-percentile of birth weight among all fetuses was 3550 g. Moreover, the proportion of LGA infants in the EmCS group (23.2%) was significantly higher than that in the NVD group (9.7%); the difference was statistically significant (*P* < .001). There were 4403 female infants (48.7%) and 4324 male infants (51.3%) in 8427 parturients. The proportion of male infants in the EmCS group (56.3%) was significantly higher than that in the NVD group (50.7%), and the difference was statistically significant. In addition, there were significant differences between the EmCS and NVD groups in terms of amniotic fluid characteristics, umbilical cord status, HDP, amniotic fluid volume, and induced labor (*P* < .001). All data are provided in Table 1.

### 3.2. The effect of maternal and infant characteristics on the risk of EmCS

Univariate analysis was performed for all variables. In all groups, the risk of EmCS increased with age (*P* < .001). Prepregnancy BMI, GWG, abnormal amniotic fluid characteristics, abnormal umbilical cord, HDP, and abnormal quantity of amniotic fluid were high-risk factors for EmCS. Multivariate logistic regression analysis was performed for variables found to significantly increase the risk of EmCS in the univariate analysis (Table 2). We found that compared with women in the younger group (<25 years old), the risk of EmCS increased with age (*P* < .001 for all). All abnormal characteristics of amniotic fluid, abnormal umbilical cord, HDP, and abnormal quantity of amniotic fluid were risk factors for an increased risk of EmCS (*P* < .05). Notably, the risk of EmCS was significantly increased (odds ratio [OR], 4.834 [ 95% confidence interval (CI), 4.093–5.710; *P* < .001) when labor induction was required. However, the risk of EmCS was not affected by fetal sex (OR, 1.096 [95% CI, 0.942–1.275]; *P* = .237).

**Table 2:** Logistic regression analyses of factors associated with EmCS (n = 8427).

Variables	Number of EmCS (%)	Univariate		Multivariate	
		OR (95% CI)	*P* value	OR (95% CI)	*P* value
Maternal age, yr					
<25	71 (6.1)	Ref		Ref	
25–29	559 (10.4)	1.771 (1.371–2.286)	<.001	1.747 (1.335–2.287)	<.001
30–34	250 (14.3)	2.538 (1.928–3.341)	<.001	2.494 (1.862–3.342)	<.001
≥35	29 (20.9)	4.025 (2.505–6.468)	<.001	4.904 (2.951–8.149)	<.001
Prepregnancy BMI groups					
Normal weight	607 (10.8)	Ref		Ref	
Underweight	206 (9.3)	0.847 (0.717–1.000)	.050	0.900 (0.751–1.079)	.256
Overweight/obese	96 (16.2)	1.602 (1.267–2.025)	<.001	1.623 (1.252–2.103)	<.001
GWG groups					
Adequate	209 (7.5)	Ref		Ref	
Inadequate	156 (12.4)	1.748 (1.404–2.177)	<.001	2.079 (1.638–2.638)	<.001
Excessive	544 (12.38)	1.740 (1.472–2.056)	<.001	1.391 (1.158–1.670)	<.001
Fetal growth					
AGA	618 (10.2)	Ref		Ref	
SGA	80 (5.5)	0.517 (0.406–0.657)	<.001	0.553 (0.426–0.719)	<.001
LGA	211 (22.4)	2.531 (2.126–3.013)	<.001	1.652 (1.306–2.090)	<.001
Characters of amniotic fluid					
Clear	438 (7.3)	Ref		Ref	
Abnormal	471 (19.6)	3.100 (2.695–3.566)	<.001	3.037 (2.612–3.531	<.001
Umbilical cord					
Normal	482 (9.2)	Ref		Ref	
Abnormal	427 (13.5)	1.541 (1.342–1.770)	<.001	1.426 (1.226–1.658)	<.001
HDP					
No	847 (10.5)	Ref		Ref	
Yes	62 (17.0)	1.749 (1.319–2.320)	<.001	1.417 (1.027–1.954)	.034
Abnormal quantity of amniotic fluid					
No	867 (9.8)	Ref		Ref	
Yes	42 (47.7)	7.869 (5.149–12.026)	<.001	7.639 (4.694–12.433)	<.001
Neonatal weight					
<3550	501 (8.2)	Ref		Ref	
≥3550	408 (17.9)	2.453 (2.131–2.823)	<.001	1.626 (1.338–1.976)	<.001
Gender					
Female	397 (9.7)	Ref		Ref	
Male	512 (11.8)	1.254 (1.091–1.440)	<.001	1.096 (0.942–1.275)	.237
Induction of labor					
No	579 (7.9)	Ref		Ref	
Yes	330 (29.0)	4.733 (4.059–5.519)	<.001	4.834 (4.093–5.710)	<.001

AGA = appropriate for gestational age, BMI = body mass index, CI = confidence interval, EmCS = emergency cesarean section, GWG = gestational weight gain, HDP = hypertensive disorders of pregnancy, LGA = large for gestational age, OR = odds ratio, Ref = reference category, SGA = small for gestational age.

Compared with pregnant women with normal prepregnancy BMI, the risk of EmCS in overweight pregnant women before pregnancy increased significantly (OR, 1.623 [95% CI, 1.252–2.103]; *P* < .001), but the risk of EmCS in low-weight pregnant women with low weight before pregnancy did not decrease (*P* = .256). Compared with adequate GWG, inadequate GWG (OR, 2.079 [95% CI, 1.638–2.638]; *P* < .001) increased the risk of EmCS to a greater extent than GWG (OR, 1.391 [95% CI, 1.158–1.670]; *P* < .001). Compared with appropriate for gestational age, SGA reduced the risk of EmCS (OR, 0.553 [95% CI, 0.426–0.719]; *P* < .001), whereas LGA increased the risk of EmCS (OR, 1.652 [95% CI, 1.306–2.090]; *P* < .001). Thus, GWG and fetal growth were still independent risk factors for EmCS, even after excluding confounding factors; however, the impact of prepregnancy maternal weight on EmCS remains to be further analyzed.

### 3.3. Subgroup analysis of risk factors based on prepregnancy BMI

We further independently analyzed pregnant women who were underweight before pregnancy (n = 2215; Table 1, Supplemental Digital Content, http://links.lww.com/MD/O275), normal weight before pregnancy (n = 5621; Table 2, Supplemental Digital Content, http://links.lww.com/MD/O275), and overweight before pregnancy (n = 591; Table 3, Supplemental Digital Content, http://links.lww.com/MD/O275) and explored whether there were differences in risk factors affecting EmCS based on different prepregnancy BMIs.

There were some similar results among all subgroups regardless of the prepregnancy BMI. The incidence of EmCS increases with increasing age. Compared with pregnant women under 25 years of age, women aged 30 to 34 years had a significantly higher risk of EmCS (*P* < .05). Compared with those with clear amniotic fluid, pregnant women with abnormal amniotic fluid had a significantly higher risk of developing EmCS (*P* < .05). Compared with newborns weighing <3550 g, those weighing >3550 g had an increased risk of EmCS (*P* < .05). Regardless of prepregnancy BMI, if pregnant women need the induction of labor, they should be alert to a significant increase in the risk of EmCS (*P* < .05), whereas fetal sex did not affect the risk of EmCS (*P* > .05).

Among pregnant women with prepregnancy underweight and normal weight, old age, inadequate GWG, LGA, and abnormal amniotic fluid volume were independent high-risk factors for EmCS (*P* < .05), whereas SGA was a low-risk factor (*P* < .05). However, if pregnant women were overweight, these factors were not independent high-risk factors, leading to an increased EmCS risk (*P* > .05). An abnormal umbilical cord was a high-risk factor for EmCS in pregnant women with normal weight (*P* < .001) and overweight (*P* = .023) but not in underweight pregnant women (*P* = .945). In the second part of this study, it was observed that pregnant women aged 25 to 29 years, GWG overdose, and HDP had an increased risk of EmCS, mainly because of the effect of normal pregnancy weight. All data are presented in Table 3.

**Table 3:** Analysis of influencing factors based on different prenatal weights.

Variables	Total (n = 8427)		Prepregnancy BMI groups					
			Underweight (n = 2215)		Normal weight (n = 5621)		Overweight (n = 591)	
	OR (95% CI)	*P* value	OR (95% CI)	*P* value	OR (95% CI)	*P* value	OR (95% CI)	*P* value
Maternal age, yr								
<25	Ref		Ref		Ref		Ref	
25–29	1.747 (1.335–2.287)	<.001	1.490 (0.916–2.424)	.108	1.802 (1.277–2.544)	.001	2.466 (0.890–6.834)	.083
30–34	2.494 (1.862–3.342)	<.001	2.102 (1.198–3.689)	.010	2.620 (1.812–3.789)	<.001	3.285 (1.128–9.569)	.029
≥35	4.904 (2.951–8.149)	<.001	5.170 (1.896–14.098)	.001	5.126 (2.768–9.494)	<.001	1.495 (0.136–16.383)	.742
GWG groups								
Adequate	Ref		Ref		Ref		Ref	
Inadequate	2.079 (1.638–2.638)	<.001	1.939 (1.270–2.966)	.002	2.194 (1.634–2.946)	<.001	7.053 (0.420–123.788)	.181
Excessive	1.391 (1.158–1.670)	<.001	0.980 (0.687–1.397)	.910	1.573 (1.254–1.973)	<.001	1.809 (0.820–3.988)	.142
Fetal growth								
AGA	Ref		Ref		Ref		Ref	
SGA	0.553 (0.426–0.719)	<.001	0.370 (0.204–0.672)	.001	0.622 (0.457–0.845)	.002	0.695 (0.258–1.869)	.471
LGA	1.652 (1.306–2.090)	<.001	1.655 (1.033–2.653)	.036	1.876 (1.394–2.527)	<.001	0.691 (0.334–1.428)	.318
Characters of amniotic fluid								
Clear	Ref		Ref		Ref		Ref	
Abnormal	3.037 (2.612–3.531)	<.001	2.952 (2.160–4.034)	<.001	2.868 (2.382–3.452)	<.001	4.768 (2.906–7.821)	<.001
Umbilical cord								
Normal	Ref		Ref		Ref		Ref	
Abnormal	1.426 (1.226–1.658)	<.001	0.989 (0.717–1.364)	.945	1.583 (1.316–1.906)	<.001	1.755 (1.079–2.855)	.023
HDP								
No	Ref		Ref		Ref		Ref	
Yes	1.417 (1.027–1.954)	.034	1.791 (0.869–3.691)	.115	1.598 (1.073–2.381)	.021	0.612 (0.247–1.517)	.289
Abnormal quantity of amniotic fluid								
No	Ref		Ref		Ref		Ref	
Yes	7.639 (4.694–12.433)	<.001	7.064 (2.815–17.725)	<.001	7.701 (4.310–13.762)	<.001	9.790–12.00	.999
Neonatal weight								
<3550	Ref		Ref		Ref		Ref	
≥3550	1.626 (1.338–1.976)	<.001	1.707 (1.157–2.518)	.007	1.554 (1.212–1.992)	<.001	1.828 (1.022–3.272)	.042
Gender								
Female	Ref		Ref		Ref		Ref	
Male	1.096 (0.942–1.275)	.237	1.075 (0.785–1.453)	.652	1.057 (0.877–1.273)	.560	1.514 (0.917–2.500)	.105
Induction of labor								
No	Ref		Ref		Ref		Ref	
Yes	4.834 (4.093–5.710)	<.001	4.138 (2.940–5.825)	<.001	5.272 (4.309–6.451)	<.001	4.059 (2.137–7.710)	<.001

AGA = appropriate for gestational age, BMI = body mass index, CI = confidence interval, GWG = gestational weight gain, HDP = hypertensive disorders of pregnancy, LGA = large for gestational age, OR = odds ratio, Ref = reference category, SGA = small for gestational age.

### 3.4. Direct causes of EmCS

Among 909 pregnant women with EmCS, the direct causes leading to the failure of VD to achieve EmCS included FD (n = 610, 67.1%), abnormal fetal position (139, 15.3%), relative cephalopelvic disproportion (n = 76, 8.4%), social factors (n = 60, 6%), others (n = 17, 1.9%), and antepartum hemorrhage (n = 7, 0.8%). Others included preeclampsia in 8 (0.88%) cases, threatened uterine rupture in 8 (0.9%) cases, and presentation of umbilical cord in 1 (0.1%) case. Among all the pregnant women who underwent EmCS, the emergency rate with medical indications was 93.4%. All women were divided into 2 groups based on the cause of EmCS: FD and nonfetal distress (NFD). The direct causes of the increased risk of EmCS caused by the abovementioned risk factors were further analyzed (Table 4). In the FD group, there were 5 cases and 1 case with an Apgar score of <8 points at 1 and 5 minutes, respectively. In the NFD group, there was 1 case and 0 cases with an Apgar score of <8 points at 1 and 5 minutes, respectively. There were no neonatal deaths in this study, and the results showed that the main cause of EmCS was NFD in the excessive GWG group (*P* = .027). The main cause of EmCS related to abnormal amniotic fluid and umbilical cord was FD, which was statistically significant (*P* < .05). Compared with the FD group, the weight of newborns in the NFD group was significantly higher (*P* < .001), and the main cause of EmCS in women with macrosomia, LGA, or birth weight ≥ 3550 g was FD (*P* < .05). For pregnant women who needed labor induction, the proportion of EmCS due to NFD after entering labor was significantly higher, and the difference was statistically significant (*P* < .001). The incidence of EmCS caused by abnormal prepregnancy weight, HDP, and amniotic fluid quantity showed no obvious tendency (*P* > .05; Table 4).

**Table 4:** Direct causes of EmCS in pregnant women.

Variables	Study population (n = 909; %)	Groups		
		Fetal distress (n = 610; 67.1%)	Nonfetal distress (n = 299; 32.9%)	*P* value
Maternal age, yr				
<25	71 (7.8)	47 (7.7)	24 (8.0)	.991
25–29	559 (61.5)	374 (61.3)	185 (61.9)	
30–35	250 (27.5)	169 (27.7)	81 (32.4)	
≥35	29 (3.2)	20 (3.3)	9 (31.0)	
BMI before pregnancy groups, kg/m^2^				
Underweight	607 (66.8)	405 (66.4)	202 (67.6)	.837
Normal weight	206 (22.7)	138 (22.6)	68 (22.7)	
Overweight/obese	96 (10.6)	67 (11.0)	29 (9.7)	
GWG groups				
Inadequate	156 (17.2)	119 (19.5)	37 (12.4)	.027
Adequate	209 (23.0)	135 (22.1)	74 (24.7)	
Excessive	544 (59.8)	356 (58.4)	188 (62.9)	
Birth weight, g	3478.41 + 387.81	3427.43 + 381.85	3582.44 + 379.59	<.001
Fetal growth				
SGA	80 (8.8)	67 (11.0)	13 (4.3)	<.001
AGA	618 (68.0)	428 (70.2)	190 (63.5)	
LGA	211 (23.2)	115 (18.9)	96 (32.1)	
Characters of amniotic fluid				
Clear	438 (48.2)	212 (34.8)	226 (75.6)	<.001
Abnormal	471 (51.8)	398 (65.2)	73 (24.4)	
Umbilical cord				
Normal	482 (53.0)	302 (49.5)	180 (60.2)	.002
Abnormal	427 (47.9)	308 (50.5)	119 (39.8)	
HDP				
No	847 (93.2)	572 (93.8)	275 (92.0)	.313
Yes	62 (6.8)	38 (6.2)	24 (8.0)	
Abnormal quantity of amniotic fluid				
No	867 (95.4)	586 (96.1)	281 (94%)	.159
Yes	42 (4.6)	24 (3.9)	18 (6.0)	
Birth weight groups				
<3550	501	364 (72.7)	137 (27.3)	<.001
≥3550	408	246 (40.3)	162 (54.2)	
Induction of labor				
No	579 (63.7)	427 (70.0)	152 (50.8)	
Yes	330 (36.3)	183 (30.0)	147 (49.2)	<.001

AGA = appropriate for gestational age, BMI = body mass index, EmCS = emergency cesarean section, GWG = gestational weight gain, HDP = hypertensive disorders of pregnancy, LGA = large for gestational age, SGA = small for gestational age.

## 4. Discussion

With the increase in pregnant women’s weight worldwide, it is still an important issue of perinatal health care to study the impact of maternal and infant weight on perinatal outcomes and provide reasonable perinatal weight management strategies.^[23,24]^ In this study, we compared the differences in various characteristics between pregnant women with NVD and those with EmCS for VD failure. We focused on the factors of maternal and fetal weight associated with the risk of EmCS and discussed the effects of different factors on the risk of EmCS based on different prepregnancy BMI states.

Abnormal maternal and fetal weights result in many long-term adverse outcomes such as diabetes mellitus and cardiovascular disease, which has become a new challenge in maternal and child healthcare.^[25,26]^ The CS rate is the main evaluation index of pregnancy outcomes associated with abnormal weight; it is also an adverse outcome that needs to be controlled.^[27]^ Although reasonable CS can protect the safety of the mother and infant, it may lead to a series of complications after surgery.^[28,29]^ Some studies have shown that mothers who are overweight before pregnancy are prone to have severe HDP, preeclampsia, or overweight GWG due to poor weight control during pregnancy, which eventually results in macrosomia or uncontrollable GDM.^[30,31]^ When these complications occur, pregnant women must terminate their pregnancies using CS. An increasing number of studies have explored reasonable perinatal weight management strategies to effectively reduce the CS rate.^[32,33]^

In addition to those with severe complications and identified indications for CS, many women still fail VD and have to undergo EmCS.^[14,34]^ Compared with those with identified ElCS indications, these patients look healthy but cannot successfully complete VD. They face more complications and greater risks in EmCS.^[17]^ Some studies have shown that the overall fetal complication rate is significantly higher in EmCS than in ElCS. Neonatal adverse outcomes increased mainly in emergency operative deliveries, and children delivered by EmCS had an increased risk of ulcerative colitis and celiac disease.^[35]^ Thus, early recognition and referral of mothers who would undergo EmCS may decrease fetal complications.^[36]^ There is already evidence that advanced age is a potential high-risk factor for EmCS; however, whether abnormal maternal and fetal weight are independent factors affecting EmCS requires further study.^[16]^

This study evaluated the impact of maternal and fetal weight on EmCS using several variables including BMI before pregnancy, BMI at delivery, GWG, SGA, and LGA. To explore the variables that predict the potential risk of EmCS, variables such as the age of the pregnant women, induction of labor, and pregnancy complications were synergistically analyzed. GWG and fetal weight were still the main factors affecting EmCS, which was consistent with other studies.^[37,38]^ However, the effect of prepregnancy weight on EmCS requires further analysis. Compared with a normal BMI, low prepregnancy weight did not reduce the risk of EmCS, whereas overweight significantly increased the risk of EmCS. Therefore, we further analyzed the risk factors of prepregnancy weight subgroups and found that regardless of the prepregnancy BMI, women with risk factors, including 30 to 34 years old, abnormal amniotic fluid, fetal weight ≥ 3550 g, and induction of labor must be vigilant against the risk of EmCS; overweight was the main risk factor for EmCS, and weight management should be strengthened during the process of pregnancy.

Direct causes of EmCS include fetal and maternal factors.^[39]^ Several studies have reported FD and relative cephalopelvic disproportion.^[40]^ Wang et al^[41]^ found that male fetus SGA with a nuchal cord has a significantly higher risk of FD during labor. In this study, factors related to fetal weight mainly caused NFD and increased the risk of EmCS, whereas other factors, such as amniotic fluid and umbilical cord, mainly caused FD and increased the risk of EmCS. However, maternal factors, such as prenatal weight and HDP, showed no obvious tendency to cause EmCS. These results suggest that the risk of EmCS should be noted in the process of VD in pregnant women with the abovementioned high-risk factors, and possible abnormalities of mothers and infants should be predicted. Once abnormalities occur in mothers and infants, EmCS should be performed within a limited time to avoid serious complications for mothers and infants.^[42,43]^ A limitation of this study was that we did not explore the influence of maternal and fetal weight on the complications of EmCS. Although the results of this study were obtained based on real-world clinical data and are reliable, they have not yet been validated using external data.

## 5. Conclusions

This study showed that maternal and fetal weight can affect the risk of EmCS. The risk factors for EmCS differed with different prepregnancy weights, and the direct factors leading to EmCS also differed. The results of this study could serve as evidence to guide pregnant women in strengthening the control of GWG in clinical practice and help obstetricians accurately predict the risk of VD failure based on their prepregnancy BMI and GWG. Further large-scale studies are required to verify these prospects.

## Author contributions

**Data curation:** Jing Bao

**Investigation:** Jing Bao, Ping Guan

**Methodology:** Jing Bao, Ping Guan

**Writing – original draft:** Jing Bao, Ping Guan

**Conceptualization:** Ping Guan

**Formal analysis:** Ping Guan

**Validation:** Ping Guan

**Writing – review & editing:** Ping Guan

## Supplementary Material



## References

[1] Yanovski JA. Obesity: trends in underweight and obesity - scale of the problem. Nat Rev Endocrinol. 2018;14:5–6. 29170540 10.1038/nrendo.2017.157 PMC5800307

[2] Muzzioli L Frigerio F Mazziotta M Donini LM Pinto A Poggiogalle E. Food compass and the challenge of sustainability on the route towards healthful diets. Sci Rep. 2024;14:6919. 38519527 10.1038/s41598-024-57615-9 PMC10960031

[3] Retnakaran R. Diabetes in pregnancy 100 years after the discovery of insulin: hot topics and open questions to be addressed in the coming years. Metabolism. 2021;119:154772. 33838145 10.1016/j.metabol.2021.154772

[4] Guan P Tang F Sun G Ren W. Prediction of emergency cesarean section by measurable maternal and fetal characteristics. J Investig Med. 2020;68:799–806. 10.1136/jim-2019-001175 PMC7057850 31980540

[5] Lin L Huang Y Chen L. Gestational weight trajectory and risk of adverse pregnancy outcomes among women with gestational diabetes mellitus: a retrospective cohort study. Matern Child Nutr. 2024;20:e13645. 38517119 10.1111/mcn.13645 PMC11168372

[6] Zong XN Li H Zhang YQ Wu HH. Reference values and growth curves of weight/length, body mass index, and ponderal index of Chinese newborns of different gestational ages. Zhonghua Er Ke Za Zhi. 2021;59:181–8. 33657691 10.3760/cma.j.cn112140-20201130-01063

[7] Ferrara A Hedderson MM Brown SD. A telehealth lifestyle intervention to reduce excess gestational weight gain in pregnant women with overweight or obesity (GLOW): a randomised, parallel-group, controlled trial. Lancet Diabetes Endocrinol. 2020;8:490–500. 32445736 10.1016/S2213-8587(20)30107-8 PMC8886506

[8] Schiavone MJ Pérez MP Aquieri A. The role of obesity in the development of preeclampsia. Curr Hypertens Rep. 2024;26:247–58. 38512586 10.1007/s11906-024-01299-z

[9] Chien P. Global rising rates of caesarean sections. BJOG. 2021;128:781–2. 33667015 10.1111/1471-0528.16666

[10] Boerma T Ronsmans C Melesse DY. Global epidemiology of use of and disparities in caesarean sections. Lancet. 2018;392:1341–8. 30322584 10.1016/S0140-6736(18)31928-7

[11] Kamel RA Negm SM Youssef A. Predicting cesarean delivery for failure to progress as an outcome of labor induction in term singleton pregnancy. Am J Obstet Gynecol. 2021;224:609.e1–609.e11. 10.1016/j.ajog.2020.12.1212 33412128

[12] Opiyo N Torloni MR Robson M. WHO’s Robson platform for data-sharing on caesarean section rates. Bull World Health Organ. 2022;100:352–4. 35521038 10.2471/BLT.21.287742 PMC9047423

[13] Beaudrot ME Elchert JA DeFranco EA. Influence of gestational weight gain and BMI on cesarean delivery risk in adolescent pregnancies. J Perinatol. 2016;36:612–7. 27054845 10.1038/jp.2016.61

[14] Muhammad T Srivastava S Kumar P Rashmi R. Prevalence and predictors of elective and emergency caesarean delivery among reproductive-aged women in Bangladesh: evidence from demographic and health survey, 2017-18. BMC Pregnancy Childbirth. 2022;22:512. 35751112 10.1186/s12884-022-04833-6 PMC9229123

[15] Sandie AB Mutua MK Sidze E. Epidemiology of emergency and elective caesarean section and its association with early neonatal mortality in sub-Saharan African countries. BMJ Open. 2023;13:e074995. 10.1136/bmjopen-2023-074995 PMC10582852 37827732

[16] Kim SY Park JY Bak SE. Effect of maternal age on emergency cesarean section. J Matern Fetal Neonatal Med. 2019;33:3969–76. 30905245 10.1080/14767058.2019.1593958

[17] Yang XJ Sun SS. Comparison of maternal and fetal complications in elective and emergency cesarean section: a systematic review and meta-analysis. Arch Gynecol Obstet. 2017;296:503–12. 28681107 10.1007/s00404-017-4445-2

[18] Heller G Bauer E Schill S. Decision-to-delivery time and perinatal complications in emergency cesarean section. Dtsch Arztebl Int. 2017;114:589–96. 28927497 10.3238/arztebl.2017.0589 PMC5615394

[19] Medicine Io. Weight Gain During Pregnancy: Reexamining the Guidelines. *In* Weight Gain During Pregnancy: Reexamining the Guidelines. Rasmussen K.M. Yaktine A.L., eds, Washington (DC). 2009:1–845. 20669500

[20] Guan P Tang F Sun G Ren W. Effect of maternal weight gain according to the Institute of Medicine recommendations on pregnancy outcomes in a Chinese population. J Int Med Res. 2019;47:4397–412. 31342872 10.1177/0300060519861463 PMC6753580

[21] Yang XQ Yang HX. Understanding and management of meconiumstained amniotic fluid during labor. Chin J Pract Gynecol Obstet. 2024;40:133–8.

[22] Zhu L Zhang R Zhang S; Chinese Neonatal Network. Chinese neonatal birth weight curve for different gestational age. Zhonghua Er Ke Za Zhi. 2015;53:97–103. 25876683

[23] Rogozinska E Zamora J Marlin N; International Weight Management in Pregnancy (i-WIP) Collaborative Group. Gestational weight gain outside the Institute of Medicine recommendations and adverse pregnancy outcomes: analysis using individual participant data from randomised trials. BMC Pregnancy Childbirth. 2019;19:322. 31477075 10.1186/s12884-019-2472-7 PMC6719382

[24] Sun Y Shen Z Zhan Y. Investigation of optimal gestational weight gain based on the occurrence of adverse pregnancy outcomes for Chinese women: a prospective cohort study. Reprod Biol Endocrinol. 2021;19:130. 34461936 10.1186/s12958-021-00797-y PMC8404327

[25] Goldstein RF Abell SK Ranasinha S. Association of gestational weight gain with maternal and infant outcomes: a systematic review and meta-analysis. JAMA. 2017;317:2207–25. 28586887 10.1001/jama.2017.3635 PMC5815056

[26] Papandreou D Mantzorou M Tyrovolas S. Pre-pregnancy excess weight association with maternal sociodemographic, anthropometric and lifestyle factors and maternal perinatal outcomes. Nutrients. 2022;14:3810. 36145183 10.3390/nu14183810 PMC9502514

[27] Xia X Zhou Z Shen S. Effect of a two-stage intervention package on the cesarean section rate in Guangzhou, China: a before-and-after study. PLoS Med. 2019;16:e1002846. 31283770 10.1371/journal.pmed.1002846 PMC6613675

[28] Rosenberg KR Trevathan WR. Evolutionary perspectives on cesarean section. Evol Med Public Health. 2018;1:67–81.

[29] Kearns RJ Shaw M Gromski PS. Neonatal and early childhood outcomes following maternal anesthesia for cesarean section: a population-based cohort study. Reg Anesth Pain Med. 2021;46:482–9. 33832987 10.1136/rapm-2020-102441

[30] Schenkelaars N Rousian M Hoek J Schoenmakers S Willemsen S Steegers-Theunissen R. Preconceptional maternal weight loss and hypertensive disorders in pregnancy: a systematic review and meta-analysis. Eur J Clin Nutr. 2021;75:1684–97. 33837274 10.1038/s41430-021-00902-9

[31] Zhang CX Lai JQ Liu KY. Optimal gestational weight gain in Chinese pregnant women by Chinese-specific body mass index categories: a multicenter prospective cohort study. Public Health Nutr. 2021;24:3210–20. 33843557 10.1017/S1368980021001622 PMC10195362

[32] Chuang FC Huang HY Chen YH Huang JP. Optimal gestational weight gain in Taiwan: a retrospective cohort study. Taiwan J Obstet Gynecol. 2024;63:220–4. 38485318 10.1016/j.tjog.2024.01.034

[33] Huang L Zhang J Sun H. Association of gestational weight gain with cesarean section: a prospective birth cohort study in Southwest China. BMC Pregnancy Childbirth. 2021;21:57. 33446128 10.1186/s12884-020-03527-1 PMC7807892

[34] Papoutsis D Antonakou A Gornall A Tzavara C Mohajer M. The SaTH risk-assessment tool for the prediction of emergency cesarean section in women having induction of labor for all indications: a large-cohort based study. Arch Gynecol Obstet. 2017;295:59–66. 27671013 10.1007/s00404-016-4209-4

[35] Darnal N Dangal G. Maternal and fetal outcome in emergency versus elective caesarean section. J Nepal Health Res Counc. 2020;18:186–9. 32969374 10.33314/jnhrc.v18i2.2093

[36] Kitaw TM Limenh SK Chekole FA Getie SA Gemeda BN Engda AS. Decision to delivery interval and associated factors for emergency cesarean section: a cross-sectional study. BMC Pregnancy Childbirth. 2021;21:224. 33743626 10.1186/s12884-021-03706-8 PMC7981954

[37] Vaananen AJ Kainu JP Eriksson H Lang M Tekay A Sarvela J. Does obesity complicate regional anesthesia and result in longer decision to delivery time for emergency cesarean section? Acta Anaesthesiol Scand. 2017;61:609–18. 28417459 10.1111/aas.12891

[38] Zhao RF Zhang WY Zhou L. Relationship between the risk of emergency cesarean section for nullipara with the prepregnancy body mass index or gestational weight gain. Zhonghua Fu Chan Ke Za Zhi. 2017;52:757–64. 29179271 10.3760/cma.j.issn.0529-567X.2017.11.008

[39] Gosset M Ilenko A Bouyou J Renevier B. Emergency caesarean section. J Visc Surg. 2017;154:47–50. 28162986 10.1016/j.jviscsurg.2016.09.012

[40] Liabsuetrakul T Sukmanee J Thungthong J Lumbiganon P. Trend of cesarean section rates and correlations with adverse maternal and neonatal outcomes: a secondary analysis of Thai universal coverage scheme data. AJP Rep. 2019;9:e328–36. 31673478 10.1055/s-0039-1697656 PMC6821536

[41] Wang L Kuromaki K Kawabe A Kikugawa A Matsunaga S Takagi A. Nuchal cord complication in male small for gestational age increases fetal distress risk during labor. Taiwan J Obstet Gynecol. 2016;55:568–74. 27590384 10.1016/j.tjog.2016.03.002

[42] Hussein BA Damtew BS Abdi HB Gudayu TW. Decision to delivery time and its predictors among mothers who underwent emergency cesarean delivery at selected hospitals of Northwest Ethiopia, 2023: Prospective Cohort Study. Int J Womens Health. 2024;16:249–64. 38352193 10.2147/IJWH.S436755 PMC10863470

[43] Damtew BS Gudayu TW Temesgan WZ Hailu AM. Effect of decision-to-delivery time of emergency cesarean section on adverse newborn outcomes at East Gojjam Zone Public Hospital, Ethiopia, March 2023: Multicenter Prospective Observational Study Design. Int J Womens Health. 2024;16:433–50. 38469355 10.2147/IJWH.S451101 PMC10926860

