# Supplementary material for: The effect of maternal and fetal weight on the risk of emergency cesarean section in nulliparous women
Source: Medicine (Baltimore). 2025 Jan 17;104(3):e41095. doi: 10.1097/MD.0000000000041095 (PMC11749654; doi:10.1097/MD.0000000000041095)
Supplement: Supplementary file 1 [file medi-104-e41095-s001.docx]

| **Supplementary Table 1**. Univariate and multivariate logistic regression analyses of factors associated with EmCS in pre-pregnancy underweight group (n=2215). | | | | | | | | |  |
| --- | --- | --- | --- | --- | --- | --- | --- | --- | --- |
| Variables | Number (n=2215) | Number of EmCS (%) | Univariate | |  | Multivariate | | | |
|  |  |  | OR (95%CI) | *p* value |  | OR (95%CI) | | *p* value | |
| Maternal age (yeaers) | |  |  |  |  |  | |  | |
| <25 | 350 | 23 (6.6) | Ref |  |  | Ref | |  | |
| 25-29 | 1443 | 132 (9.1) | 1.431 (0.904-2.266) | 0.126 |  | 1.490 (0.916-2.424) | | 0.108 | |
| 30-34 | 389 | 44 (11.3) | 1.813 (1.071-3.070) | 0.027 |  | 2.102 (1.198-3.689) | | 0.010 | |
| ≥35 | 33 | 7 (21.2) | 3.828 (1.502-9.756) | 0.005 |  | 5.170 (1.896-14.098) | | 0.001 | |
| GWG groups |  |  |  |  |  |  | |  | |
| Adequate | 870 | 68 (7.8) | Ref |  |  | Ref | |  | |
| Inadequate | 377 | 49 (13.0) | 1.762 (1.194-2.600) | 0.004 |  | 1.939 (1.270-2.966) | | 0.002 | |
| Excessive | 89 | 968 (9.2) | 1.194 (0.859-1.661) | 0.292 |  | 0.980 (0.687-1.397) | | 0.910 | |
| Fetal growth |  |  |  |  |  |  | |  | |
| AGA | 1571 | 143 (9.1) |  |  |  |  | |  | |
| SGA | 412 | 14 (3.4) | 0.351 (0.201-0.615) | <0.001 |  | 0.370 (0.204-0.672) | | 0.001 | |
| LGA | 232 | 49 (21.1) | 2.674 (1.867-3.829) | <0.001 |  | 1.655 (1.033-2.653) | | 0.036 | |
| Characters of amniotic fluid | | |  |  |  |  | | | |
| Clear | 1636 | 105 (6.4) | Ref |  |  | Ref | |  | |
| Abnormal | 579 | 101 (17.4) | 3.081(2.301-4.125) | <0.001 |  | 2.952 (2.160-4.034) | | <0.001 | |
| Umbilical cord | |  |  |  |  |  | |  | |
| Normal | 1402 | 126 (9.0) | Ref |  |  | Ref | |  | |
| Abnormal | 813 | 80 (9.8) | 1.105 (0.823-1.484) | 0.505 |  | 0.989 （0.717-1.364） | | 0.945 | |
| HDP |  |  |  |  |  |  | |  | |
| No | 2152 | 193 (9.0) |  |  |  |  | |  | |
| Yes | 63 | 13 (20.6) | 2.639 (1.409-4.944) | 0.002 |  | 1.791 (0.869-3.691) | | 0.115 | |
| Abnormal quantity of amniotic fluid | | | |  |  |  | | | |
| No | 2191 | 195 (8.9) | Ref |  |  | Ref | |  | |
| Yes | 24 | 11 (45.8) | 8.661 (3.829-19.592) | <0.001 |  | 7.064 (2.815-17.725) | | <0.001 | |
| Neonatal weight | |  |  |  |  |  | | | |
| <3550 | 1618 | 107 (6.6) | Ref |  |  | Ref | |  | |
| ≥3550 | 597 | 99 (16.6) | 2.807 (2.097-3.757) | <0.001 |  | 1.707 (1.157-2.518) | | 0.007 | |
| Gender |  |  |  |  |  |  | |  | |
| Female | 1059 | 88 (8.3) | Ref |  |  | | Ref |  | |
| Male | 1156 | 118 (10.2) | 1.254 (0.939-1.676) | 0.125 |  | | 1.075 (0.785-1.453) | 0.652 | |
| Induction of labor | |  |  |  |  |  | |  | |
| No | 1907 | 132 (6.9) | Ref |  |  | Ref | |  | |
| Yes | 308 | 74 (24.0) | 4.252 (3.102-5.830) | <0.001 |  | 4.138 ()2.940-5.825 | | <0.001 | |
| **Abbreviations**: EmCS: emergency cesarean section; OR: odds ratio; CI: confidence intervals; Ref: reference category; GWG: gestational weight gain; AGA: appropriate for gestational age; SGA: small for gestational age; LGA: large for gestational age; HDP: hypertensive disorders of pregnancy. | | | | | | | | |  |

| **Supplementary Table 2**. Univariate and multivariate logistic regression analyses of factors associated with EmCS in pre-pregnancy normal weight group (n=5621). | | | | | | | | |
| --- | --- | --- | --- | --- | --- | --- | --- | --- |
| Variables | Number (n=5621) | Number of EmCS (%) | Univariate | |  | Multivariate | | |
|  |  |  | OR (95%CI) | *p value* |  | OR (95%CI) | | *p value* |
| Maternal age (years) | |  |  |  |  |  | |  |
| <25 | 735 | 43 (5.9) | Ref |  |  | Ref | |  |
| 25-29 | 3579 | 366 (10.2) | 1.833 (1.323-2.541) | <0.001 |  | 1.802 (1.277-2.544) | | 0.001 |
| 30-34 | 1211 | 177 (14.6) | 2.755 (1.974-3.897) | <0.001 |  | 2.620 (1.812-3.789) | | <0.001 |
| ≥35 | 96 | 21 (21.9) | 4.506 (2.539-7.997) | <0.001 |  | 5.126 (2.768-9.494) | | <0.001 |
| GWG groups |  |  |  |  |  |  | |  |
| Adequate | 1832 | 131 (7.2) | Ref |  |  | Ref | |  |
| Inadequate | 874 | 106 (12.1) | 1.792 (1.368-2.347) | <0.001 |  | 2.194 (1.634-2.946) | | <0.001 |
| Excessive | 2915 | 370 (12.7) | 1.888 (1.532-2.325) | <0.001 |  | 1.573 (1.254-1.973) | | <0.001 |
| Fetal growth |  |  |  |  |  |  | |  |
| AGA | 4048 | 403 (10.0) | Ref |  |  | Ref | |  |
| SGA | 957 | 60 (6.3) | 0.605 (0.457-0.801) | <0.001 |  | 0.622 (0.457-0.845) | | 0.002 |
| LGA | 616 | 144 (23.4) | 2.759 (2.230-3.415) | <0.001 |  | 1.876 (1.394-2.527) | | <0.001 |
| Characters of amniotic fluid | | |  |  |  |  | | |
| Clear | 3961 | 293 (7.4) | Ref |  |  | Ref | |  |
| Abnormal | 1660 | 314 (18.9) | 2.920 (2.461-3.465) | <0.001 |  | 2.868 (2.382-3.452) | | <0.001 |
| Umbilical cord | |  |  |  |  |  | |  |
| Normal | 3499 | 310 (8.9) | Ref |  |  | Ref | |  |
| Abnormal | 2122 | 297 (14.0) | 1.674 (1.413-1.983) | <0.001 |  | 1.583 (1.316-1.906) | | <0.001 |
| HDP |  |  |  |  |  |  | |  |
| No | 5388 | 565 (10.5) | Ref |  |  | Ref | |  |
| Yes | 233 | 42 (18.0) | 1.877 (1.329-2.651) | <0.001 |  | 1.598 (1.073-2.381) | | 0.021 |
| Abnormal quantity of amniotic fluid | | | |  |  |  | | |
| No | 5559 | 578 (10.4) | Ref |  |  | Ref | |  |
| Yes | 62 | 29 (46.8) | 7.537 (4.565-12.564) | <0.001 |  | 7.701 (4.310-13.762) | | <0.001 |
| Neonatal weight | |  |  |  |  |  | | |
| <3550 | 4164 | 346 (8.3) | Ref | <0.001 |  | Ref | |  |
| ≥3550 | 1457 | 261 (17.9) | 2.408 (2.025-2.864) |  |  | 1.554 (1.212-1.992) | | <0.001 |
| Gender |  |  |  |  |  |  | |  |
| Female | 2769 | 270 (9.8) | Ref |  |  | | Ref |  |
| Male | 2852 | 337 (11.8) | 1.240 (1.047-1.469) | 0.013 |  | | 1.057 (0.877-1.273) | 0.560 |
| Induction of labor | |  |  |  |  |  | |  |
| No | 4859 | 374 (7.7) | Ref |  |  | Ref | |  |
| Yes | 762 | 233 (30.6) | 5.282 (4.382-6.366) | <0.001 |  | 5.272 (4.309-6.451) | | <0.001 |
| **Abbreviations**: EmCS: emergency cesarean section; OR: odds ratio; CI: confidence intervals; Ref: reference category; GWG: gestational weight gain; AGA: appropriate for gestational age; SGA: small for gestational age; LGA: large for gestational age; HDP: hypertensive disorders of pregnancy. | | | | | | | | |

| **Supplementary Table 3.** Univariate and multivariate logistic regression analyses of factors associated with EmCS in pre-pregnancy overweight group (n=591). | | | | | | | | |
| --- | --- | --- | --- | --- | --- | --- | --- | --- |
| Variables | Number (n=591 | Number of EmCS (%) | Univariate | |  | Multivariate | | |
|  |  |  | OR (95%CI) | *p value* |  | OR (95%CI) | | *p value* |
| Maternal age (years) | |  |  |  |  |  | |  |
| <25 | 5 | 5 (7.1) |  |  |  |  | |  |
| 25-29 | 357 | 61 (17.1) | 2.679 (1.036-6.930) | 0.042 |  | 2.466 (0.890-6.834) | | 0.083 |
| 30-34 | 154 | 29 (18.8) | 3.016 (1.115-8.160) | 0.030 |  | 3.285 (1.128-9.569) | | 0.029 |
| ≥35 | 10 | 1 (10.0) | 1.444 (0.151-13.806) | 0.750 |  | 1.495 (0.136-16.383) | | 0.742 |
| GWG groups |  |  |  |  |  |  | |  |
| Adequate | 79 | 10 (13.4) |  |  |  |  | |  |
| Inadequate | 3 | 1 (33.3) | 3.450 (0.286-41.627) | 0.330 |  | 7.053 (0.420-123.788) | | 0.181 |
| Excessive | 509 | 85 (16.7) | 1.383 (0.685-2.794) | 0.366 |  | 1.809 (0.820-3.988) | | 0.142 |
| Fetal growth |  |  |  |  |  |  | |  |
| AGA | 426 | 72 (16.9) |  |  |  |  | |  |
| SGA | 70 | 6 (8.6) | 0.461 (0.192-1.105) | 0.083 |  | 0.695 (0.258-1.869) | | 0.471 |
| LGA | 95 | 18 (18.9) | 1.149 (0.649-2.037) | 0.634 |  | 0.691 (0.334-1.428) | | 0.318 |
| Characters of amniotic fluid | | |  |  |  |  | | |
| Clear | 423 | 40 (9.5) |  |  |  |  | |  |
| Abnormal^*^ | 168 | 56 (33.3) | 4.787 (3.031-7.562) | <0.001 |  | 4.768 (2.906-7.821) | | <0.001 |
| Umbilical cord | |  |  |  |  |  | |  |
| Normal | 355 | 46 (13.0) |  |  |  |  | |  |
| Abnormal | 236 | 50 (21.2) | 1,806 (1.163-2.803) | 0.008 |  | 1.755 (1.079-2.855) | | 0.023 |
| HDP |  |  |  |  |  |  | |  |
| No | 823 | 89 (17.0) |  |  |  |  | |  |
| Yes | 68 | 7 (10.3) | 0.560 (0.248-1.264) | 0.163 |  | 0.612 (0.247-1.517) | | 0.289 |
| Abnormal quantity of amniotic fluid | | | |  |  |  | | |
| No | 589 | 94 (16.0) |  |  |  |  | |  |
| Yes | 2 | 2 (100) | 8.57 (0-10.000) | 0.999 |  | 9.790-12.000 | | 0.999 |
| Neonatal weight | |  |  |  |  |  | | |
| <3550 | 363 | 48 (13.2) |  |  |  |  | |  |
| ≥3550 | 228 | 48 (21.1) | 1.750 (1.127-2.717) | 0.013 |  | 1.828 (1.022-3.272) | | 0.042 |
| Gender |  |  |  |  |  |  | |  |
| Female | 275 | 39 (14.2) |  |  |  | |  |  |
| Male | 316 | 57 (18.0) | 1.332 (0.854-2.076) | 0.206 |  | | 1.514 (0.917-2.500) | 0.105 |
| Induction of labor | |  |  |  |  |  | |  |
| No | 523 | 73 (14.0) |  |  |  |  | |  |
| Yes | 68 | 23 (33.8) | 3.151 (1.800-5.516) | <0.001 |  | 4.059 (2.137-7.710) | | <0.001 |
| **Abbreviations**: EmCS: emergency cesarean section; OR: odds ratio; CI: confidence intervals; Ref: reference category; GWG: gestational weight gain; AGA: appropriate for gestational age; SGA: small for gestational age; LGA: large for gestational age; HDP: hypertensive disorders of pregnancy. | | | | | | | | |
